# Comparison of detector performance in small 6 MV and 6 MV FFF beams using a Versa HD accelerator

**DOI:** 10.1371/journal.pone.0213253

**Published:** 2019-03-11

**Authors:** Paula Monasor Denia, María del Carmen Castellet García, Carla Manjón García, Juan David Quirós Higueras, Noelia de Marco Blancas, Jorge Bonaque Alandí, Xavier Jordi Juan Senabre, Agustín Santos Serra, Juan López-Tarjuelo

**Affiliations:** Servicio de Radiofísica y Protección Radiológica, Consorcio Hospitalario Provincial de Castellón, Castellón de la Plana, España; North Shore Long Island Jewish Health System, UNITED STATES

## Abstract

**1. Background & purpose:**

Investigate the applicability of a series of detectors in small field dosimetry and the possible differences between their responses to FF and FFF beams. This work extends upon the series of detectors used by other authors to also include metal-oxide-semiconductor field-effect transistors (MOSFETs) detectors and radiochromic film. We also included a later correction of output factors (OFs) recommended by the recently published IAEA´s code of practice TRS 483 on dosimetry of small static fields used in external beam radiotherapy.

**2. Materials & methods:**

The OFs, profiles, and PDDs of 6 MV and 6 MV FFF beams were measured with 11 different detectors using field sizes between 0.6 × 0.6 cm^2^ and 10 × 10 cm^2^.

**3. Results:**

The OFs of the FFF beams were lower than those of the FF beams for field sizes larger than 3 × 3 cm^2^ but higher for field sizes smaller than 3 × 3 cm^2^. After applying the IAEA´s TRS 483 corrections, the final OFs were compatible with our initial results when considering uncertainties involved. Small-volume detectors are preferable for measuring the penumbra of these small fields where this attribute is higher in the crossline direction than in the inline direction. The R_100_ of equivalent-quality FFF beams was higher compared to the corresponding flattened beams.

**4. Conclusions:**

We observed no difference for the dose responses between 6 MV and 6 MV FFF beams for any of the detectors. OF results, profiles and PDDs were clearly consistent with the previously published literature regarding the Versa HD linac. Correcting our first OFs, taken as ratio of detector charges, with the IAEA´s TRS 483 corrections to obtain the final OFs, did not make the former significantly different.

## Introduction

With technological advances there has been an increase in the use of techniques such as static and dynamic intensity-modulated radiotherapy, volumetric modulated arc therapy, and stereotactic cranial and extra-cranial radiotherapy, as well as the use of flattening filter-free (FFF) beams [1–3]. These techniques have the common characteristic of using fields and small segments to maximally optimise patient treatments by varying the fluence without requiring homogeneous flat beams.

Recent studies based on measurements [4–6] and Monte Carlo simulations [7,8] for Elekta linacs [9–12] show the characteristics and advantages of FFF beams over flattening filter (FF) beams, including the highest dose rates, reduced head scattering, less leakage, and smaller out-of-field doses, among others. Thus, non-standard FFF beams with small fields have become the object of interest and study.

Small fields are characterised by loss of lateral charged-particle equilibrium [13–18]. Moreover, the perturbation correction factors for these particles are difficult to calculate [15]. This results in deviations from Bragg-Gray cavity theory and a lack of balance in the detectors because these have a finite size. Some authors separate these perturbation factors into those caused by volume effects and those due to the difference between the density of the detector and water [19–21].

Numerous authors have evaluated these perturbation factors for diodes, diamond detectors, and ionisation chambers (ICs) for small fields using the Monte Carlo method [21–27]. There are also numerous experimental studies on the response of these detectors [13, 14, 18, 21, 28, 29], but few of these include FFF beams because they are more commonly used for CyberKnife applications [22, 28, 30]. The International Atomic Energy Agency (IAEA) code of practice TRS 483, published at the end of 2017 by the time we were preparing this manuscript, collects all these perturbation factors [18].

This study aimed to investigate the applicability of a series of detectors in small field dosimetry and the possible differences between their responses to FF and FFF beams in order to increase the data available to users regarding their characterisation. In this regard, readers should consider the diverse range of equipment used by medical physics services: although these are limited, staff managing them may need additional references to compare their measurements.

In the case of output factors (OFs), we have extended the range of detectors used to include metal-oxide-semiconductor field-effect transistors (MOSFETs) and radiochromic film. First, in the absence of a clear consensus regarding small field OF determination, they were obtained directly as a ratio of detector charge readouts, also considering a daisy-chaining approach [31]. However, with the publication of the corrections recommended by IAEA's code of practice TRS 483 to derive output factors as quotients of absorbed doses [18], we have been able to investigate the agreement between both sets of data. It should not be overlooked that the selection of data among these kinds of sets as an input for planning therapy systems has been posing a critical decision for medical physicists involved in clinical calculations.

## Materials and methods

We used a Versa HD linear accelerator (Elekta, Stockholm, Sweden) equipped with 6 MV and 6 MV FFF energy beams and an Agility head (Elekta, Stockholm, Sweden) with a multileaf collimator (MLC) with 160 leaves of 5-mm thick interdigitation-capable tungsten projected into the isocenter. The sheets move at a maximum speed of 3.5 cm/s and the MLC does not have a backup jaw. Its dose rate at 6 MV can reach 600 UM/min and this reaches up to 1400 UM/min with the 6 MV FFF beam. The remaining geometric and dosimetric properties of the Versa HD accelerator are described elsewhere [32–35]. The beam quality for the 6 MV beam is a tissue phantom ratio (TPR)_20/10_ of 0.684 and the TPR_20/10_ for the 6 MV FFF beam is 0.674. The accelerator was calibrated to administer 1 cGy/MU at a 10-cm depth in water, for a 10 × 10 cm^2^ field, and at a source-to-surface distance of 90 cm.

To compare the detectors, OF measurements, profiles (inline and crossline), and percentage depth dose (PDD) measurements for fields between 0.6 × 0.6 cm^2^ and 10 × 10 cm^2^ were taken. The following detectors (whose main characteristics can be found in Table 1), were used: Gafchromic EBT3 radiochromic film (Ashland Advanced Materials, Bridgewater, USA), TN-502RDM-H reinforced mobile microMOSFET (Best Medical, Ottawa, Canada), electron field detector (EFD; Scanditronix Medical AB, Uppsala, Sweden), stereotactic field detector (SFD; Scanditronix Wellhöfer AB, Uppsala, Sweden), photon field detector (PFD-3G; IBA Dosimetry AB, Uppsala, Sweden), microDiamond diamond detector (PTW, Freiburg, Germany), PinPoint 3D IC (PTW, Freiburg, Germany), Semiflex 3D IC (PTW, Freiburg, Germany), CC13-S IC (IBA Dosimetry, Germany), FC65-G Farmer IC (IBA Dosimetry, Germany), and a PPC40 IC (IBA Dosimetry, Germany).

**Table 1:** Characteristics of the different detectors.

	Type	Active volume (mm^3^)	Material
**FC65-G**	Air ionisation chamber	650	Graphite and Aluminium
**CC13-S**	Air ionisation chamber	130	PEEK and C-552
**Semiflex 3D**	Air ionisation chamber	70	PMMA, Graphite and Aluminium
**PinPoint**	Air ionisation chamber	16	PMMA, Graphite and Aluminium
**MicroDiamond**	Synthetic diamond	0,004	Diamond
**SFD**	Unshielded diode	0,017	Silicon
**EFD**	Unshielded diode	0,188	Silicon
**MOSFET**	Metal oxide semiconductor field effect transistor	2 · 10^−5^	SiO_2_ and Silicon
**EBT3**	Radiochromic film	N/A	Active layer based on diacetylene monomers with polyester coating

The OFs were measured with 9 different detectors for field sizes between 0.6 × 0.6 cm^2^ and 10 × 10 cm^2^ at a 10-cm depth on the PTW BeamScan water phantom (PTW, Freiburg, Germany) under isocentric conditions (source-to-surface distance = 90 cm) and with the DOSE 1 electrometer (IBA Dosimetry, Germany). The TRUFIX system from PTW was used to place the microDiamond detector and the diodes at their effective points with the axis of symmetry parallel to the radiation beam. The ICs were also placed using the TRUFIX system at their geometric centres with their axes of symmetry perpendicular to the radiation beam and parallel to the movement of the leaves. The microMOSFETs and radiochromic films were centred (visually) in the luminous field and perpendicular to the radiation beam following the TRS 483 recommendations [18].

OFs were measured with radiochromic film by placing pieces of the film between blocks of plastic water (CIRS, Norfolk, VA, USA) at a depth of 10 cm. They were then scanned, processed, and analysed with the web application for radiochromic film dosimetry found at http://www.Radiochromic.com (Radiochromic S.L., Girona, Spain). The OFs were calculated as the average of five 200 MU measurements, corrected for the pressure and temperature for each IC. The measurements presented were normalised to 3 × 3 cm^2^ of the OF (the smallest field in which the lateral charged-particle equilibrium was sufficient for both energies).

A ‘reference detector’—the average of the most suitable detectors for each field size—was considered for each field size. This was obtained by comparing our results with the available literature published on the different detectors and with the recommendations for use provided by the manufacturers. The differences between the OFs obtained with the different detectors and those from the reference detectors were calculated.

Parallel to this study, we performed the OFs corrections recommended in the TRS 483 for four of the detectors used in this work. In addition to this we obtained an estimate for OFs with CC13-S results, given that CC13-S and CC13 are built in a close way and the code of practice only reports corrections for the latter model.

The TRS 483 denotes the output correction factor with $k_{Q_{clin},Q_{msr}}^{f_{clin}{,f}_{msr}}$ and states that it be applied to the OFs in the following way:

$${OF}_{Q_{clin}{,Q}_{msr}}^{f_{clin},f_{msr}}=\frac{M_{Q_{clin}}^{f_{clin}}}{M_{Q_{msr}}^{f_{msr}}}k_{Q_{clin}{,Q}_{msr}}^{f_{clin},f_{msr}}$$ (1)

being $\frac{M_{Q_{clin}}^{f_{clin}}}{M_{Q_{msr}}^{f_{msr}}}$ the ratio of detector readings in water (corrected for influence quantities) in the clinical field *f*clin with beam quality *Q*clin and in the machine specific reference field *f*msr with beam quality *Q*msr. It should be noted here that TRS 483 uses the symbol ${Ω}_{Q_{clin}{,Q}_{msr}}^{f_{clin},f_{msr}}$ instead of ${OF}_{Q_{clin}{,Q}_{msr}}^{f_{clin}{,f}_{msr}}$; but we keep this last because it is more familiar to the readership.

Our OFs are presented in this case normalized to 10 x 10 cm^2^ in order to apply the correction factors of TRS 483, for field sizes between 0.6 × 0.6 cm^2^ and 4 × 4 cm^2^ at a 10-cm depth too.

Along with OFs, we present the experimental uncertainty associated to the ratio of detector readings and also its combination to the uncertainty given in Table 37 of TRS 483 for $k_{Q_{clin},Q_{msr}}^{f_{clin},f_{msr}}$ to get the uncertainty for OFs.

Moreover, the most critical field OFs (0.6 × 0.6 cm^2^ and 1 × 1 cm^2^) were also studied by means of daisy chaining [36] in 2 x 2 cm^2^, 3 x 3 cm^2^, and 4 x 4 cm^2^. In this situation OFs are obtained as:

$${OF}_{Q_{clin}{,Q}_{msr}}^{f_{clin},f_{msr}}=\frac{M_{Q_{clin}}^{f_{clin}}}{M_{Q_{int}}^{f_{int}}}\frac{M_{Q_{int}}^{f_{int}}}{M_{Q_{msr}}^{f_{msr}}}\frac{k_{Q_{clin}{,Q}_{msr}}^{f_{clin},f_{msr}}}{k_{Q_{int}{,Q}_{msr}}^{f_{int},f_{msr}}}k_{Q_{int}{,Q}_{msr}}^{f_{int}{,f}_{msr}}={OF^{\prime }}_{Q_{clin}{,Q}_{int}}^{f_{clin}{,f}_{int}}\;{OF^{\prime }}_{Q_{int}{,Q}_{msr}}^{f_{int}{,f}_{msr}}\frac{k_{Q_{clin},Q_{msr}}^{f_{clin},f_{msr}}}{k_{Q_{int}{,Q}_{msr}}^{f_{int}{,f}_{msr}}}k_{Q_{int}{,Q}_{msr}}^{f_{int},f_{msr}}$$ (2)

where *int* denotes the intermediate square field used for daisy chaining.

For the profiles and PDDs, we used the PTW TRUFIX system, placing the ICs parallel to the radiation beam for the profiles and in the perpendicular direction for the PDDs. The detectors and diodes were oriented with the axis parallel to the beam so that their sensitive volume was perpendicular to it, both for the profiles and for the PDDs. Both the crossline and inline profiles, were measured for 6 MV and 6 MV FFF beams and for field sizes between 0.6 × 0.6 cm^2^ and 10 × 10 cm^2^ at 5 different depths (16, 50, 100, 200, and 300 mm) in the PTW BeamScan water phantom (PTW, Freiburg, Germany) under isocentric conditions (source-to-surface distance = 90 cm) and with the PTW MEPHYSTO mc^2^ acquisition system (PTW, Freiburg, Germany). The acquisition mode was continuous with a speed of 2 mm/s and a resolution of 0.5 mm. The field size, penumbra size, flatness, and symmetry were also recorded.

The PDDs were measured with the same equipment and methods as the profiles, from a depth of 30 cm. For the PDDs, the depth of the dose maximum and that of 50% of the absorbed dose (R_100_ and R_50_) values, normalised to the maximum dose distance (d_max_) are presented. For the analysis, we used the PTW Analize software (PTW, Freiburg, Germany) for averaging, interpolation, and smoothing of the curves. In the same way as for the OFs, a ‘reference detector’ was marked for the profiles and PDDs based on our results, previous publications, and recommendations for use, which in our opinion, represents the most appropriate detector for each measurement at each field size.

## Results

### Outputs factors

The bottom of Tables 2 and 3 show the differences between the detectors with respect to the OF references (shaded entries). As shown by these results, some ICs are not suitable for use with certain field sizes. In other words, where the detector’s active volume is the same or a greater order of magnitude than the size of the OF to be measured. For example, the FC65-G camera is not suitable for measuring the OF of 0.6 × 0.6 cm^2^, as shown by its 70% difference with respect to the reference OF.

**Table 2:** Output factors for different detectors depending on the field size for 6 MV beams.

	OUTPUT FACTORS								
**Field size (cm**^**2**^**)**	**0.6 x 0.6**		**1 x 1**	**2 x 2**	**3 x 3**	**4 x 4**	**5 x 5**	**7 x 7**	**10 x 10**
**FC65-G**		0.143	0.368	0.780	1.000	1.068	1.104	1.157	1.220
**CC13-S**		0.325	0.680	0.939	1.000	1.041	1.074	1.127	1.184
**Semiflex 3D**		0.380	0.718	0.940	1.000	1.038	1.071	1.123	1.182
**PinPoint**		0.445	0.746	0.942	1.000	1.040	1.072	1.124	1.178
**MicroDiamond**		0.518	0.794	0.950	1.000	1.038	1.069	1.121	1.181
**SFD**		0.522	0.786	0.945	1.000	1.042	1.076	1.136	1.203
**EFD**		0.503	0.789	0.950	1.000	1.038	1.070	1.125	1.203
**MOSFET**		0.520	0.793	0.945	1.000	1.041	1.073	1.145	1.202
**EBT3**		0.505	0.793	0.941	1.000	1.040	1.091	1.141	1.169
		DIFFERENCES							
**Reference Output Factor**		**0.516**	**0.791**	**0.942**	**1.000**	**1.040**	**1.073**	**1.136**	**1.202**
**FC65-G**		-72.3	-53.5	-17.2	0.0	2.7	2.9	1.8	1.5
**CC13-S**		-37.0	-14.0	-0.3	0.0	0.1	0.1	-0.8	-1.5
**Semiflex 3D**		-26.4	-9.2	-0.2	0.0	-0.2	-0.2	-1.1	-1.7
**PinPoint**		-13.8	-5.7	0.0	0.0	0.0	-0.1	-1.1	-2.0
**MicroDiamond**		0.4	0.4	0.8	0.0	-0.2	-0.4	-1.3	-1.7
**SFD**		1.2	-0.6	0.3	0.0	0.2	0.3	0.0	0.1
**EFD**		-2.5	-0.3	0.8	0.0	-0.2	-0.3	-1.0	0.1
**MOSFET**		0.8	0.3	0.3	0.0	0.1	0.0	0.8	0.0
**EBT3**		-2.1	0.3	-0.1	0.0	0.0	1.7	0.4	-2.7

The shaded squares highlight the detectors used as a reference for each field size. The bottom of the table shows the difference (expressed as a percentage) between detector responses for each field size with respect to the chosen reference detector.

**Table 3:** Output factors for different detectors depending on field size for 6 MV FFF beams.

	OUTPUT FACTORS							
**Field size (cm**^**2**^**)**	**0.6 x 0.6**	**1 x 1**	**2 x 2**	**3 x 3**	**4 x 4**	**5 x 5**	**7 x 7**	**10 x 10**
**FC65-G**	0.138	0.369	0.788	1.000	1.059	1.090	1.134	1.181
**CC13-S**	0.339	0.704	0.940	1.000	1.034	1.061	1.104	1.144
**Semiflex 3D**	0.409	0.744	0.943	1.000	1.034	1.061	1.104	1.149
**PinPoint**	0.468	0.766	0.945	1.000	1.036	1.063	1.102	1.145
**MicroDiamond**	0.572	0.819	0.952	1.000	1.031	1.058	1.099	1.144
**SFD**	0.586	0.813	0.949	1.000	1.038	1.066	1.114	1.180
**EFD**	0.566	0.817	0.953	1.000	1.033	1.060	1.104	1.165
**MOSFET**	0.555	0.790	0.951	1.000	1.027	1.061	1.115	1.166
**EBT3**	0.483	0.776	0.947	1.000	1.046	1.068	1.113	1.164
	DIFFERENCES							
**Reference Output Factor**	**0.570**	**0.810**	**0.945**	**1.000**	**1.034**	**1.061**	**1.114**	**1.163**
**FC65-G**	-75.8	-54.4	-16.6	0.0	2.4	2.7	1.8	1.5
**CC13-S**	-40.5	-13.1	-0.5	0.0	0.0	0.0	-0.9	-1.6
**Semiflex 3D**	-28.2	-8.1	-0.2	0.0	0.0	0.0	-0.9	-1.2
**PinPoint**	-17.9	-5.4	0.0	0.0	0.2	0.2	-1.1	-1.5
**MicroDiamond**	0.4	1.1	0.7	0.0	-0.3	-0.3	-1.3	-1.6
**SFD**	2.8	0.4	0.4	0.0	0.4	0.5	0.0	1.5
**EFD**	-0.7	0.9	0.8	0.0	-0.1	-0.1	-0.9	0.2
**MOSFET**	-2.6	-2.5	0.6	0.0	-0.7	0.0	0.1	0.3
**EBT3**	-15.3	-4.2	0.2	0.0	1.2	0.7	-0.1	0.1

The shaded squares highlight the detectors used as a reference for each field size. The bottom of the table shows the difference (expressed as a percentage) between detector responses for each field size with respect to the chosen reference detector.

Tables 2 and 3 show that the microMOSFETs and diodes behave properly for all field sizes; the microDiamond detector responded well in fields up to 7 × 7 cm^2^, beyond which it started to underestimate the OF. The PinPoint, Semiflex 3D, CC13-S, and FC65-G ICs under-responded for the smallest fields (0.6 × 0.6 and 1 × 1 cm^2^) because they have a higher active volume. However, as reported in both Table 2 and 3, these ICs are ideal for dose measurements from larger fields, except for the PinPoint IC which underestimates the OF from 7 × 7 cm^2^ because of the central electrode effect.

The reference OFs for each field size correctly correspond with the OFs obtained with the EBT3 radiochromic film, with deviations of less than 3% for both the FF and FFF beams for all field sizes. There was no significant difference (less than 3% for all suitable detectors at each field size) in the dose responses between 6 MV and 6MV FFF beams for any of the detectors. As already demonstrated for the Versa HD by other authors [37], the OFs of FFF beams were lower than the FF beams for field sizes larger than 3 × 3 cm^2^, but higher for field sizes smaller than 3 × 3 cm^2^.

Finally, in the top of Tables 4 and 5 we show the ratio of detector readings measured directly and also with the daisy chaining in 4 x 4 cm^2^, 3 x 3 cm^2^ and 2 x 2 cm^2^ along with their corresponding uncertainties. This ratio of detector charges is what was considered as the OF before the TRS 483 publication; nevertheless this code of practice emphasizes that it is not the OF, straightforwardly defined as a quotient of absorbed doses, because the $k_{Q_{clin}{,Q}_{msr}}^{f_{clin},f_{msr}}$ is mitted or unknown. In the bottom of these tables we present the corrected OFs taking the $k_{Q_{clin},Q_{msr}}^{f_{clin},f_{msr}}$ from table 26 of TRS 483 along with their uncertainties.

**Table 4:** Ratio of detector readings for different detectors depending on the field size for 6 MV beams (top of the table) and output factors with TRS 483 correction (bottom of the table).

Field size (cm^2^)	4 x 4	3 x 3	2 x 2	1 x 1	0.6 x 0.6	4 x 4	3 x 3	2 x 2	1 x 1	0.6 x 0.6
**Detector**	**Ratio of detector charge readings**					**u (k = 2)**				
**MicroDiamond**	0.880	0.848	0.805	0.675	0.441	0.003	0.003	0.003	0.002	0.003
**SFD**	0.863	0.828	0.782	0.653	0.431	0.010	0.010	0.009	0.008	0.006
**EFD**	0.862	0.830	0.788	0.656	0.418	0.062	0.060	0.057	0.046	0.029
**PinPoint**			0.799					0.006		
**CC13-S**	0.879					0.009				
** **	**Ratio of detector charge with daisychaining in 4 cm**									
**MicroDiamond**				0.674	0.440				0.007	0.005
**SFD**				0.665	0.439				0.010	0.007
**EFD**				0.669	0.427				0.045	0.028
	**Ratio of detector charge with daisychaining in 3 cm**									
**MicroDiamond**				0.673	0.439				0.005	0.004
**SFD**				0.667	0.440				0.009	0.007
**EFD**				0.668	0.426				0.045	0.028
** **										
** **	**Ratio of detector charge with daisychaining in 2 cm**										
**MicroDiamond**				0.669	0.437				0.005	0.004
**SFD**				0.667	0.440				0.009	0.007
**EFD**				0.664	0.424				0.045	0.028
**Field size (cm**^**2**^**)**	**4 x 4**	**3 x 3**	**2 x 2**	**1 x 1**	**0.6 x 0.6**	**4 x 4**	**3 x 3**	**2 x 2**	**1 x 1**	**0.6 x 0.6**
**Detector**	**Output factor with TRS 483 correction**					**u (k = 2)**				
**MicroDiamond**	0.880	0.848	0.803	0.664	0.429	0.006	0.007	0.007	0.007	0.007
**SFD**	0.885	0.852	0.807	0.665	0.430	0.012	0.012	0.011	0.010	0.008
**EFD**	0.874	0.843	0.800	0.658	0.415	0.063	0.061	0.059	0.047	0.029
**PinPoint**			0.802					0.009		
**CC13-S**	0.880					0.011				
** **	**Output factor with TRS 483 correction with daisychaining in 4 cm**									** **
**MicroDiamond**				0.663	0.426				0.012	0.009
**SFD**				0.660	0.424				0.014	0.010
**EFD**				0.662	0.416				0.046	0.028
** **										
** **	**Output factor with TRS 483 correction with daisychaining in 3 cm**						** **			
**MicroDiamond**				0.662	0.425				0.011	0.008
**SFD**				0.660	0.423				0.013	0.010
**EFD**				0.660	0.415				0.046	0.028
** **	**Output factor with TRS 483 correction with daisychaining in 2 cm**									** **
**MicroDiamond**				0.663	0.426				0.011	0.009
**SFD**				0.660	0.424				0.013	0.010
**EFD**				0.659	0.414				0.046	0.029

The right side of the table shows the uncertainty associated with the process. Uncertainties for CC13-S are estimates because TRS 483 only reports data on CC13.

**Table 5:** Ratio of detector readings for different detectors depending on the field size for 6 FFF MV beams (top of the table) and output factors with TRS 483 correction (bottom of the table).

**Field size (cm**^**2**^**)**	**4 x 4**	**3 x 3**	**2 x 2**	**1 x 1**	**0.6 x 0.6**	** **	**4 x 4**	**3 x 3**	**2 x 2**	**1 x 1**	**0.6 x 0.6**
**Detector**	**Ratio of detector charge readings**						**u (k = 2)**				
**MicroDiamond**	0.902	0.873	0.832	0.714	0.498		0.002	0.002	0.002	0.003	0.001
**SFD**	0.870	0.848	0.803	0.682	0.490		0.012	0.004	0.003	0.008	0.008
**EFD**	0.892	0.863	0.822	0.703	0.484		0.046	0.020	0.019	0.015	0.010
**PinPoint**			0.825						0.006		
**CC13-S**	0.903						0.007				
** **	**Ratio of detector charge with daisychaining in 4 cm**						** **				** **
**MicroDiamond**				0.714	0.497					0.005	0.004
**SFD**				0.707	0.508					0.013	0.011
**EFD**				0.676	0.464					0.036	0.025
** **											
** **	**Ratio of detector charge with daisychaining in 3 cm**						** **				** **
**MicroDiamond**				0.712	0.496					0.007	0.005
**SFD**				0.700	0.503					0.010	0.010
**EFD**				0.709	0.488					0.017	0.011
** **											
** **	**Ratio of detector charge with daisychaining in 2 cm**						** **				** **
**MicroDiamond**				0.708	0.494					0.005	0.004
**SFD**				0.701	0.503					0.010	0.009
**EFD**				0.706	0.486					0.016	0.011
**Field size (cm**^**2**^**)**	**4 x 4**	**3 x 3**	**2 x 2**	**1 x 1**	**0.6 x 0.6**	** **	**4 x 4**	**3 x 3**	**2 x 2**	**1 x 1**	**0.6 x 0.6**
**Detector**	**Output factor with TRS 483 correction**						**u (k = 2)**				
**MicroDiamond**	0.902	0.873	0.830	0.702	0.482		0.004	0.005	0.005	0.011	0.002
**SFD**	0.892	0.873	0.829	0.695	0.485		0.014	0.005	0.004	0.010	0.011
**EFD**	0.904	0.877	0.835	0.705	0.478		0.047	0.020	0.020	0.015	0.010
**PinPoint**			0.828						0.009		
**CC13-S**	0.904						0.009				
** **	**Output factor with TRS 483 correction with daisychaining in 4 cm**						** **				** **
**MicroDiamond**				0.702	0.480					0.009	0.007
**SFD**				0.702	0.491					0.018	0.016
**EFD**				0.669	0.452					0.037	0.025
** **											
** **	**Output factor with TRS 483 correction with daisychaining in 3 cm**						** **				** **
**MicroDiamond**				0.700	0.480					0.016	0.010
**SFD**				0.693	0.484					0.014	0.014
**EFD**				0.701	0.475					0.017	0.011
** **											
** **	**Output factor with TRS 483 correction with daisychaining in 2 cm**						** **				** **
**MicroDiamond**				0.702	0.482					0.011	0.009
**SFD**				0.694	0.485					0.014	0.013
**EFD**				0.701	0.475					0.016	0.011

The right side of the table shows the uncertainty associated with the process. Uncertainties for CC13-S are estimates because TRS 483 only reports data on CC13.

The Tables 4 and 5 show that the uncertainty of the EFD associated with the lack of reproducibility of its reading is about 4 times greater than that of the microDiamond and the SFD for 6 MV, and slightly lower for 6 FFF MV. EFD also had an infra-response for 0.6 x 0.6 cm^2^ with both energies.

The process of correcting with TRS 483 $k_{Q_{clin}{,Q}_{msr}}^{f_{clin}{,f}_{msr}}$ convert dissimilar reading ratios to similar OFs in the case of microDiamond and SFD. Furthermore, final OFs are all compatible with every other when measurement uncertainty is taken into account, for both energies.

The daisy chaining procedure increases the experimental uncertainty by relying on more electrometer readings and seems to result in close reading ratios for microDiamond and SFD, but at the end did not lead to close OFs.

All these results are also shown in a more visual way in Figs 1–8.

**Fig 1:**
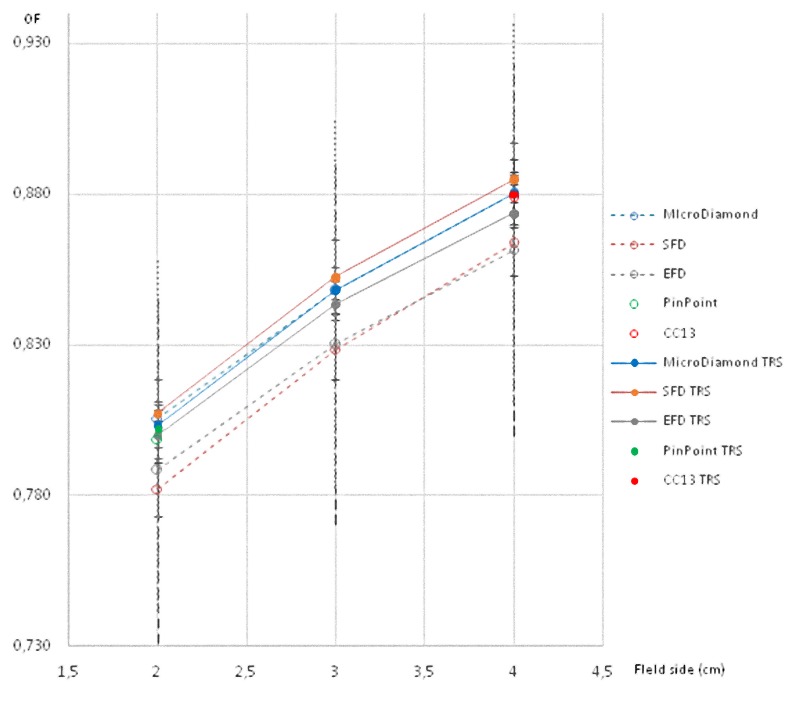
**Ratio of detector readings and output factors for the 2 × 2 cm^2^, 3 × 3 cm^2^, and 4 × 4 cm^2^ fields for the 6 MV beams**.

**Fig 2:**
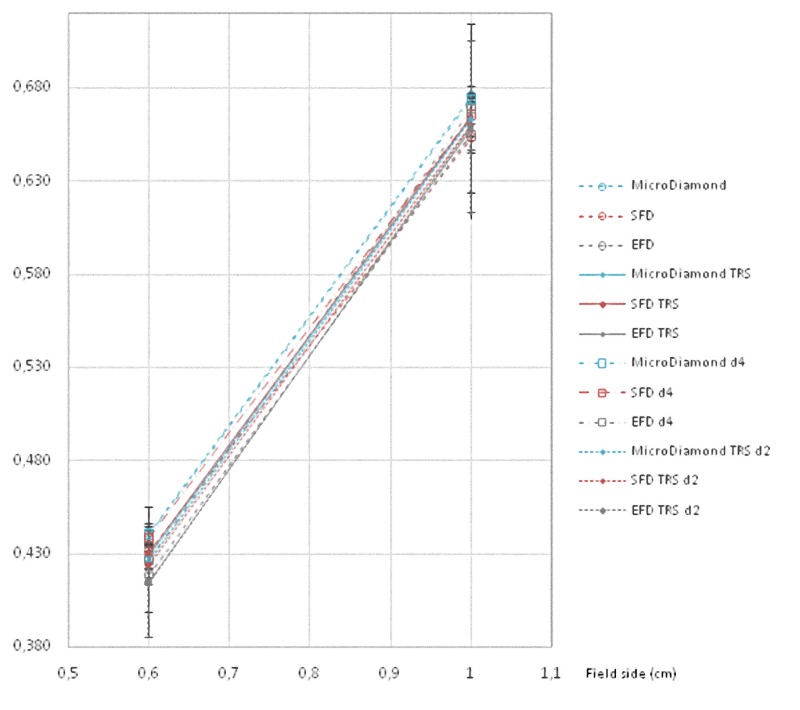
Ratio of detector readings and output factors (OFs) for the 0.6 × 0.6 cm^2^ and 1 × 1 cm^2^ fields for the 6 MV beams with different detectors, and also with daisy chaining in 4 x 4 cm^2^ and 2 x 2 cm^2^.

**Fig 3:**
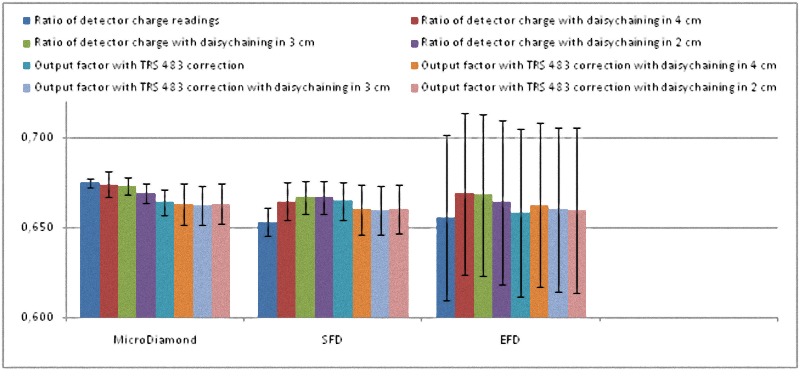
Ratio of detector readings and OFs for the 0.6 × 0.6 cm^2^ field for the 6 MV beams with different detectors. Results with daisy chaining in 4 x 4 cm^2^, 3 x 3 cm^2^, and 2 x 2 cm^2^ are included.

**Fig 4:**
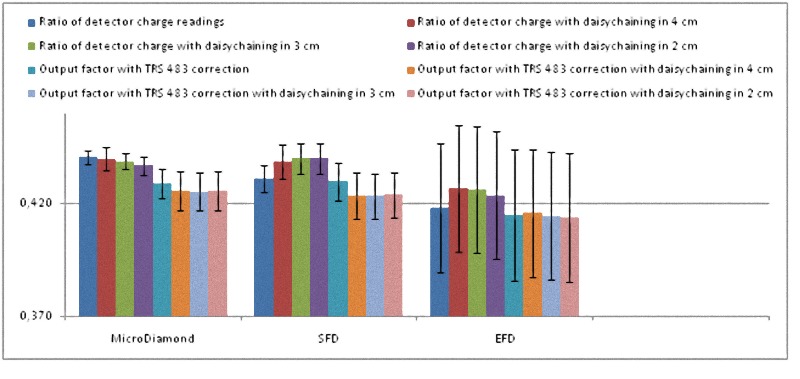
Ratio of detector readings and OFs for the 1 × 1 cm^2^ field for the 6 MV beams with different detectors. Results for daisy chaining in 4 x 4 cm^2^, 3 x 3 cm^2^, and 2 x 2 cm^2^ are included.

**Fig 5:**
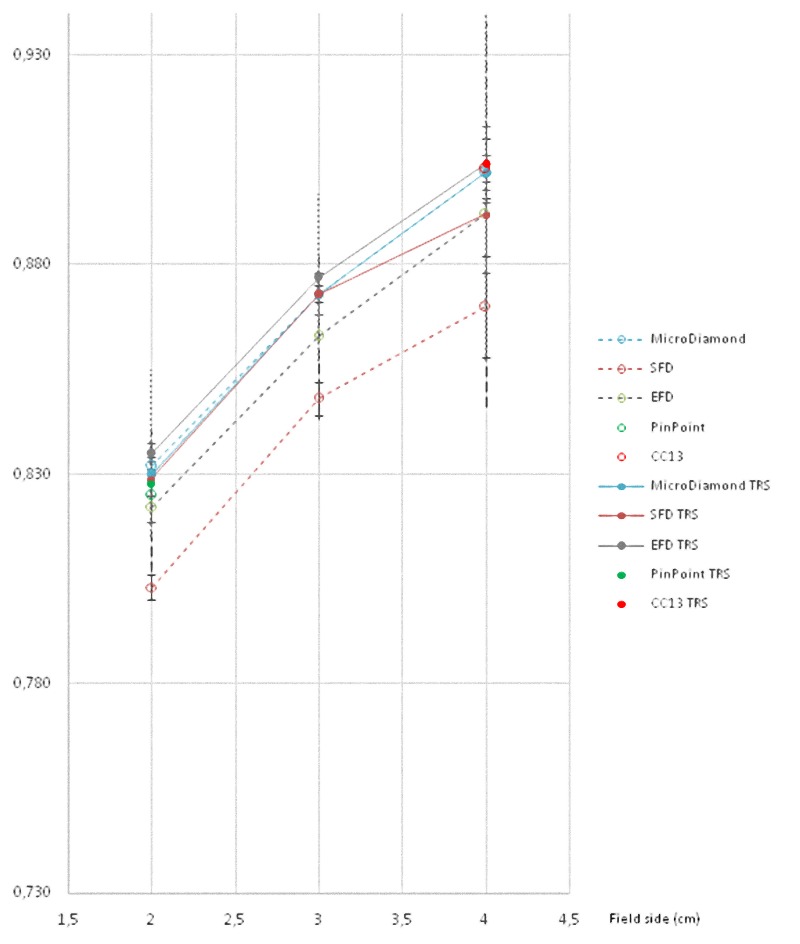
Ratio of detector readings and output factors for the 2 × 2 cm^2^, 3 × 3 cm^2^ and 4 × 4 cm^2^ fields for the 6 FFF MV beams.

**Fig 6:**
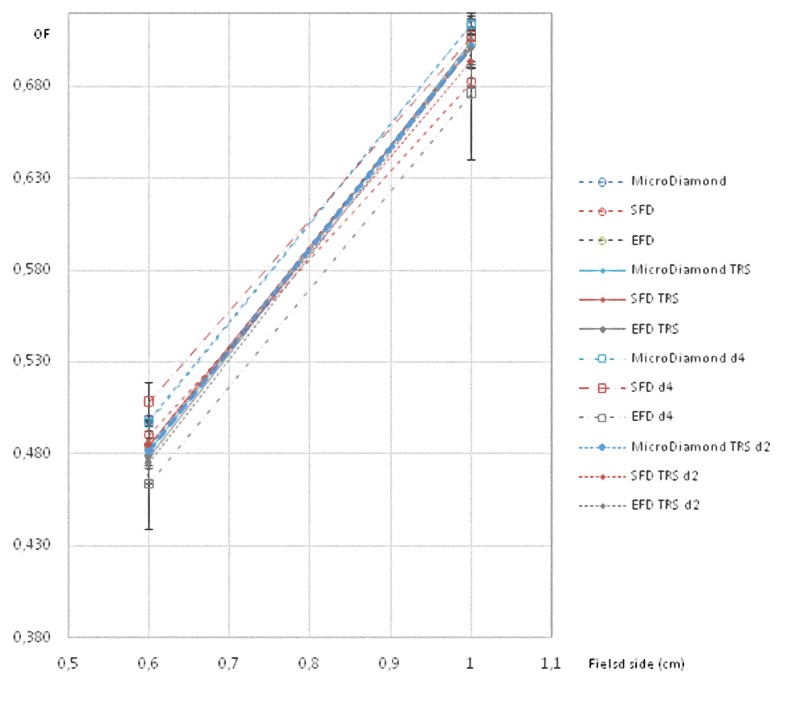
Ratio of detector readings and output factors for the 0.6 × 0.6 cm^2^ and 1 × 1 cm^2^ fields for the 6 FFF MV beams with different detectors, with daisy chaining in 4 x 4 cm^2^ without correction and in 2 x 2 cm^2^ with the TRS 483 correction.

**Fig 7:**
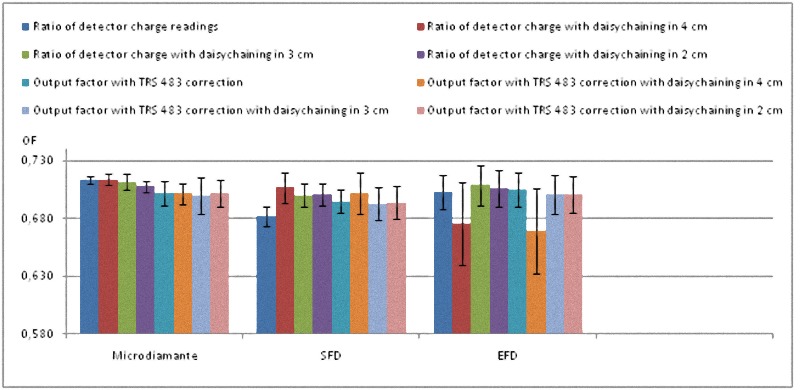
Ratio of detector readings and OFs for the 0.6 × 0.6 cm^2^ field for the 6 FFF MV beams with different detectors. Results with daisy chaining in 4 x 4 cm^2^, 3 x 3 cm^2^, and in 2 x 2 cm^2^ are also presented.

**Fig 8:**
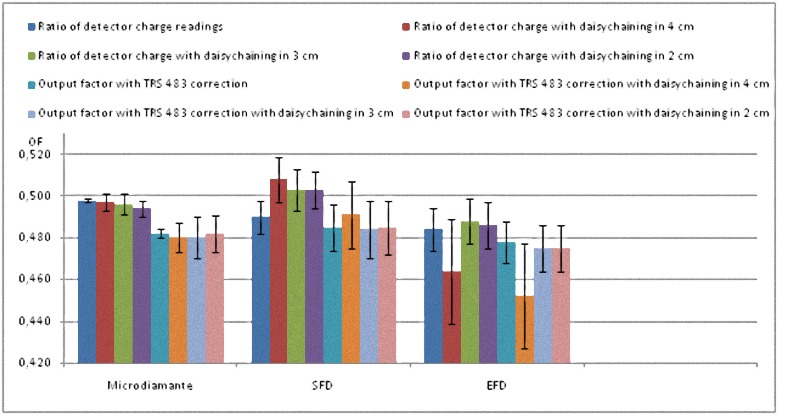
Ratio of detector readings and OFs for the 1 × 1 cm^2^ field for the 6 FFF MV beams with different detectors. Results with daisy chaining in 4 x 4 cm^2^, 3 x 3 cm^2^, and in 2 x 2 cm^2^ are also presented.

### Profiles

**Fig 9:**
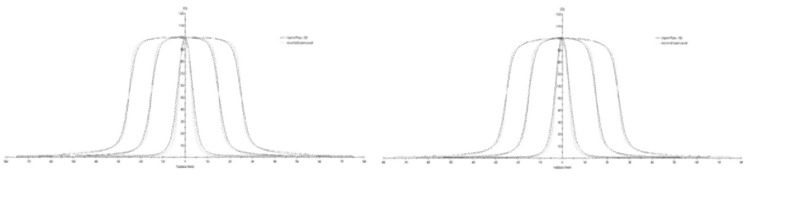
**In-plane profiles at a 100-cm depth for the 0.6 × 0.6 cm^2^, 3 × 3 cm^2^, and 5 × 5 cm^2^ fields for the 6 MV (left) and 6 MV FFF (right) beams with different detectors**.

Fig 9 shows the in-plane profiles at a 100-cm depth for the 0.6 × 0.6 cm^2^, 3 × 3 cm^2^, and 5 × 5 cm^2^ fields for the 6 MV and 6 MV FFF beams with the Semiflex 3D IC and the microDiamond detector which clearly shows that the penumbra is best characterised by the detector with the lowest active volume. For the profiles, the values for the field size, penumbra (average of the left and right penumbra), flatness (‘unflatness’ for the FFF beams), and symmetry for all the field sizes at a 100-mm depth are presented in Tables 6 and 7. The shaded entries in the tables also indicate the reference detector used to make the comparisons and calculate the deviations for each field size.

**Table 6:** Results of the crossline and inline profile parameters for different detectors at a 100-mm depth depending on the field size for 6 MV beams.

**0.6 × 0.6 cm**^**2**^	**Crossline**				**Inline**			
**6 MV**	**Field size (cm)**	**Penumbra (mm)**	**Flatness (%)**	**Symmetry (%)**	**Field size (cm)**	**Penumbra (mm)**	**Flatness (%)**	**Symmetry (%)**
**PFD-3G**	0.674	3.535	107.06	107.06	0.633	2.815	105.59	105.59
**SFD**	0.663	3.480	106.10	106.51	0.605	2.440	103.34	103.34
**MicroDiamond**	0.687	3.655	100.00	100.46	0.627	2.685	101.23	101.23
**SemiFlex 3D**	0.820	4.360	100.31	100.31	0.741	3.770	100.12	100.12
**1 × 1 cm**^**2**^	**Crossline**				**Inline**			
**6 MV**	**Field size (cm)**	**Penumbra (mm)**	**Flatness (%)**	**Symmetry (%)**	**Field size (cm)**	**Penumbra (mm)**	**Flatness (%)**	**Symmetry (%)**
**PFD-3G**	1.049	4.100	100.04	100.04	1.012	3.020	100.97	100.97
**SFD**	1.056	4.070	100.00	100.00	1.026	2.755	100.00	100.00
**MicroDiamond**	1.060	4.260	100.07	100.07	1.023	2.940	100.79	100.79
**SemiFlex 3D**	1.112	5.060	100.09	100.09	1.062	4.260	100.01	100.01
**2 × 2 cm**^**2**^	**Crossline**				**Inline**			
**6 MV**	**Field size (cm)**	**Penumbra (mm)**	**Flatness (%)**	**Symmetry (%)**	**Field size (cm)**	**Penumbra (mm)**	**Flatness (%)**	**Symmetry (%)**
**PFD-3G**	2.029	4.610	100.06	100.06	2.018	3.310	100.03	100.03
**SFD**	2.041	4.695	100.21	100.21	2.025	3.180	100.28	100.28
**MicroDiamond**	2.028	4.880	100.17	100.17	2.032	3.320	100.14	100.14
**SemiFlex 3D**	2.032	5.920	100.06	100.06	2.056	4.835	100.06	100.06
**3 × 3 cm**^**2**^	**Crossline**				**Inline**			
**6 MV**	**Field size (cm)**	**Penumbra (mm)**	**Flatness (%)**	**Symmetry (%)**	**Field size (cm)**	**Penumbra (mm)**	**Flatness (%)**	**Symmetry (%)**
**PFD-3G**	3.026	4.805	100.62	100.45	3.011	3.515	100.47	100.28
**SFD**	3.039	4.935	100.75	100.58	3.029	3.460	101.64	100.84
**MicroDiamond**	3.034	5.115	100.39	100.22	3.028	3.575	100.54	100.52
**SemiFlex 3D**	3.042	6.265	100.66	100.31	3.055	5.140	100.72	100.57
**4 × 4 cm**^**2**^	**Crossline**				**Inline**			
**6 MV**	**Field size (cm)**	**Penumbra (mm)**	**Flatness (%)**	**Symmetry (%)**	**Field size (cm)**	**Penumbra (mm)**	**Flatness (%)**	**Symmetry (%)**
**PFD-3G**	4.029	5.045	100.97	100.55	4.024	3.650	100.91	100.82
**SFD**	4.028	5.255	101.04	100.73	4.018	3.710	101.23	100.89
**MicroDiamond**	4.030	5.370	100.91	100.47	4.032	3.755	100.82	100.47
**SemiFlex 3D**	4.033	6.515	101.05	100.40	4.073	5.330	100.90	100.48
**5 × 5 cm**^**2**^	**Crossline**				**Inline**			
**6 MV**	**Field size (cm)**	**Penumbra (mm)**	**Flatness (%)**	**Symmetry (%)**	**Field size (cm)**	**Penumbra (mm)**	**Flatness (%)**	**Symmetry (%)**
**PFD-3G**	5.024	5.225	101.27	100.65	4.997	3.780	101.08	100.55
**SFD**	5.030	5.445	101.91	101.07	5.030	3.965	101.50	101.12
**MicroDiamond**	5.034	5.500	101.01	100.50	5.040	3.940	101.23	100.57
**SemiFlex 3D**	5.053	6.715	101.25	100.29	5.040	5.565	101.07	100.36
**7 × 7 cm**^**2**^	**Crossline**				**Inline**			
**6 MV**	**Field size (cm)**	**Penumbra (mm)**	**Flatness (%)**	**Symmetry (%)**	**Field size (cm)**	**Penumbra (mm)**	**Flatness (%)**	**Symmetry (%)**
**PFD-3G**	7.015	5.455	101.64	100.56	7.029	4.000	101.58	100.67
**MicroDiamond**	7.022	5.885	102.18	100.94	7.034	4.240	101.82	101.20
**SemiFlex 3D**	7.042	5.815	102.37	101.10	6.997	4.225	101.90	100.94
**10 × 10 cm**^**2**^	**Crossline**				**Inline**			
**6 MV**	**Field size (cm)**	**Penumbra (mm)**	**Flatness (%)**	**Symmetry (%)**	**Field size (cm)**	**Penumbra (mm)**	**Flatness (%)**	**Symmetry (%)**
**PFD-3G**	10.001	5.985	102.45	100.90	9.987	4.320	101.91	100.88
**MicroDiamond**	10.039	6.310	103.18	101.28	10.022	4.670	102.58	100.93
**SemiFlex 3D**	10.037	7.610	102.40	100.42	10.087	6.335	102.00	100.82

The shaded squares highlight the detectors used as a reference for each field size.

**Table 7:** Results of the crossline and inline profile parameters for different detectors at a 100-mm depth depending on the field size for 6 MV FFF beams.

**0.6 × 0.6 cm**^**2**^	**Crossline**				**Inline**			
**6 MV FFF**	**Field size (cm)**	**Penumbra (mm)**	**Flatness (%)**	**Symmetry (%)**	**Field size (cm)**	**Penumbra (mm)**	**Flatness (%)**	**Symmetry (%)**
**PFD-3G**	0.649	3.720	1.255	100.62	0.577	2.870	1.211	101.71
**SFD**	0.625	3.580	1.245	101.59	0.570	2.535	1.171	102.42
**MicroDiamond**	0.649	3.785	1.256	100.41	0.596	2.780	1.201	102.42
**SemiFlex 3D**	0.742	4.385	1.266	100.66	0.682	3.850	1.264	100.49
**1 × 1 cm**^**2**^	**Crossline**				**Inline**			
**6 MV FFF**	**Field size (cm)**	**Penumbra (mm)**	**Flatness (%)**	**Symmetry (%)**	**Field size (cm)**	**Penumbra (mm)**	**Flatness (%)**	**Symmetry (%)**
**PFD-3G**	1.012	4.405	1.192	100.58	1.002	3.165	1.101	100.49
**SFD**	1.026	4.330	1.174	100.86	0.999	2.750	1.077	100.64
**MicroDiamond**	1.025	4.485	1.188	100.68	0.990	3.000	1.089	100.45
**SemiFlex 3D**	1.053	5.175	1.232	100.45	1.032	4.395	1.190	100.42
**2 × 2 cm**^**2**^	**Crossline**				**Inline**			
**6 MV FFF**	**Field size (cm)**	**Penumbra (mm)**	**Flatness (%)**	**Symmetry (%)**	**Field size (cm)**	**Penumbra (mm)**	**Flatness (%)**	**Symmetry (%)**
**PFD-3G**	1.997	4.885	1.073	100.41	2.016	3.485	1.038	100.23
**SFD**	2.012	4.860	1.077	100.77	2.002	3.220	1.044	100.76
**MicroDiamond**	2.000	4.975	1.077	100.26	2.010	3.400	1.046	100.45
**SemiFlex 3D**	2.000	5.940	1.106	100.45	2.037	4.940	1.073	100.30
**3 × 3 cm**^**2**^	**Crossline**				**Inline**			
**6 MV FFF**	**Field size (cm)**	**Penumbra (mm)**	**Flatness (%)**	**Symmetry (%)**	**Field size (cm)**	**Penumbra (mm)**	**Flatness (%)**	**Symmetry (%)**
**PFD-3G**	3.006	5.010	1.040	100.58	3.017	3.605	1.031	100.47
**SFD**	3.024	4.995	1.042	101.07	3.009	3.425	1.032	100.65
**MicroDiamond**	3.011	5.190	1.042	100.45	3.015	3.575	1.034	100.28
**SemiFlex 3D**	3.008	6.195	1.059	100.53	3.023	5.150	1.045	100.21
**4 × 4 cm**^**2**^	**Crossline**				**Inline**			
**6 MV FFF**	**Field size (cm)**	**Penumbra (mm)**	**Flatness (%)**	**Symmetry (%)**	**Field size (cm)**	**Penumbra (mm)**	**Flatness (%)**	**Symmetry (%)**
**PFD-3G**	4.002	5.125	1.032	100.74	4.022	3.700	1.029	100.22
**SFD**	4.013	5.240	1.040	101.19	3.999	3.670	1.031	100.72
**MicroDiamond**	4.013	5.265	1.036	100.71	4.030	3.725	1.033	100.52
**SemiFlex 3D**	4.015	6.340	1.045	100.90	4.058	5.295	1.038	100.33
**5 × 5 cm**^**2**^	**Crossline**				**Inline**			
**6 MV FFF**	**Field size (cm)**	**Penumbra (mm)**	**Flatness (%)**	**Symmetry (%)**	**Field size (cm)**	**Penumbra (mm)**	**Flatness (%)**	**Symmetry (%)**
**PFD-3G**	5.009	5.185	1.040	101.18	5.034	3.800	1.030	100.31
**SFD**	5.023	5.425	1.045	101.42	5.018	3.845	1.032	100.59
**MicroDiamond**	5.012	5.380	1.040	100.96	5.035	3.860	1.033	100.43
**SemiFlex 3D**	5.025	6.445	1.043	100.78	5.061	5.415	1.034	100.24
**7 × 7 cm**^**2**^	**Crossline**				**Inline**			
**6 MV FFF**	**Field size (cm)**	**Penumbra (mm)**	**Flatness (%)**	**Symmetry (%)**	**Field size (cm)**	**Penumbra (mm)**	**Flatness (%)**	**Symmetry (%)**
**PFD-3G**	6.990	5.410	1.053	101.44	7.023	3.970	1.046	100.46
**MicroDiamond**	7.002	5.670	1.053	101.25	7.020	4.100	1.044	100.45
**SemiFlex 3D**	-	-	-	-	-	-	-	-
**10 × 10 cm**^**2**^	**Crossline**				**Inline**			
**6 MV FFF**	**Field size (cm)**	**Penumbra (mm)**	**Flatness (%)**	**Symmetry (%)**	**Field size (cm)**	**Penumbra (mm)**	**Flatness (%)**	**Symmetry (%)**
**PFD-3G**	9.993	5.710	1.151	101.78	10.016	4.245	1.132	100.47
**MicroDiamond**	10.000	6.035	1.150	101.23	10.052	4.455	1.137	100.58
**SemiFlex 3D**	-	-	-	-	-	-	-	-

The shaded squares highlight the detectors used as a reference for each field size.

These tables show that the symmetry values for the FF beams are practically equal to those for the FFF beam (within the 100–102.58% range), except for the 0.6 × 0.6 cm^2^ field size, for all the detectors. The flatness (‘unflatness’ for the FFF beams) shows the same behaviour, within the 100–103.18% range for the FF beam and 1.029–1.232% range for the ‘unflatness’ of the FFF beams.

Regarding the penumbra, for both energies and field sizes, the average penumbra in the crossline was greater than in the in-plane at around 1–1.5 mm. The Semiflex 3D IC overestimated both the field size and the penumbra for the smaller field sizes (0.6 × 0.6 cm^2^ and 1 × 1 cm^2^) compared to the other detectors. For the other field sizes (from 2 × 2 cm^2^ to 10 × 10 cm^2^), the field size measurements were within 1% of the difference with respect to each field-size reference (shaded entries in Tables 6 and 7), for both the FF and FFF beams. Finally, the Semiflex 3D IC still overestimated the penumbra for these fields size.

### Percentage depth dose

As shown in Table 8, some values for the FFF beam are a little higher than for the FF beam, both for R_100_ and R_50_. The maximum difference reached was 1.5 mm for R_100_, even though the quality of the FFF beam was matched to be the equivalent of the corresponding flattened beams, as specified in the Elekta customer acceptance test [38].

**Table 8:** Results of the parameters for different detectors depending on the field size for 6 MV and 6 MV FFF beams.

**0.6 × 0.6 cm**^**2**^	**6 MV**		**6 MV FFF**	
	**R**_**100**_**(mm)**	**R**_**50**_**(mm)**	**R**_**100**_**(mm)**	**R**_**50**_**(mm)**
**SFD**	10.51	116.50	11.50	113.04
**MicroDiamond**	10.99	115.11	11.51	115.20
**SemiFlex 3D**	12.49	130.50	11.02	126.25
**1 × 1 cm**^**2**^	**6 MV**		**6 MV FFF**	
	**R**_**100**_**(mm)**	**R**_**50**_**(mm)**	**R**_**100**_**(mm)**	**R**_**50**_**(mm)**
**SFD**	12.52	122.69	13.51	121.55
**MicroDiamond**	12.48	121.03	14.00	120.28
**SemiFlex 3D**	12.98	124.50	14.50	123.03
**2 × 2 cm**^**2**^	**6 MV**		**6 MV FFF**	
	**R**_**100**_**(mm)**	**R**_**50**_**(mm)**	**R**_**100**_**(mm)**	**R**_**50**_**(mm)**
**SFD**	14.99	127.64	15.01	128.18
**MicroDiamond**	15.02	126.49	16.02	126.37
**SemiFlex 3D**	14.50	126.09	15.51	126.47
**PinPoint**	14.49	125.91	15.03	126.12
**3 × 3 cm**^**2**^	**6 MV**		**6 MV FFF**	
	**R**_**100**_**(mm)**	**R**_**50**_**(mm)**	**R**_**100**_**(mm)**	**R**_**50**_**(mm)**
**PPC40**	13.90	131.30	16.50	131.90
**SFD**	16.50	131.49	16.51	132.23
**MicroDiamond**	15.99	130.65	16.98	130.50
**SemiFlex 3D**	15.01	129.55	16.00	129.25
**PinPoint**	14.50	128.82	16.48	129.28
**4 × 4 cm**^**2**^	**6 MV**		**6 MV FFF**	
	**R**_**100**_**(mm)**	**R**_**50**_**(mm)**	**R**_**100**_**(mm)**	**R**_**50**_**(mm)**
**PPC40**	15.49	133.17	16.01	133.15
**SFD**	15.52	136.65	16.52	136.04
**MicroDiamond**	16.51	133.62	17.00	133.78
**SemiFlex 3D**	15.01	133.06	16.48	133.31
**PinPoint**	14.50	132.37	16.00	132.73
**5 × 5 cm**^**2**^	**6 MV**		**6 MV FFF**	
	**R**_**100**_**(mm)**	**R**_**50**_**(mm)**	**R**_**100**_**(mm)**	**R**_**50**_**(mm)**
**PPC40**	14.99	136.09	16.02	135.98
**SFD**	16.00	139.33	16.01	140.23
**MicroDiamond**	15.54	137.29	16.99	136.67
**SemiFlex 3D**	14.51	136.09	16.00	136.64
**PinPoint**	15.00	135.72	15.98	135.79
**7 × 7 cm**^**2**^	**6 MV**		**6 MV FFF**	
	**R**_**100**_**(mm)**	**R**_**50**_**(mm)**	**R**_**100**_**(mm)**	**R**_**50**_**(mm)**
**PPC40**	14.53	141.67	16.01	141.54
**MicroDiamond**	16.01	142.55	17.48	142.30
**SemiFlex 3D**	14.52	141.74	16.03	141.65
**PinPoint**	14.48	141.78	16.01	140.97
**10 × 10 cm**^**2**^	**6 MV**		**6 MV FFF**	
	**R**_**100**_**(mm)**	**R**_**50**_**(mm)**	**R**_**100**_**(mm)**	**R**_**50**_**(mm)**
**PPC40**	14.99	148.67	16.50	147.34
**MicroDiamond**	15.51	149.33	16.99	148.05
**SemiFlex 3D**	15.48	149.03	15.99	147.54
**PinPoint**	14.52	148.95	16.50	147.27

The shaded squares highlight the detectors used as a reference for each field size.

The shaded entries in the Table 8 show the reference detector used for each field size. For the 0.6 × 0.6 cm^2^ and 1 × 1 cm^2^ fields the reference detector was the SFD. These measurements were not performed for the PPC40 detector because its volume is greater than these field sizes. The Semiflex 3D detector showed a maximum difference of 15% (less than 2 mm) while the maximum difference of the microDiamond was 4% (less than 0.5 mm) for both the FF and FFF beams.

For 2 × 2 cm^2^ fields, in both the FF and FFF beams, the differences for the PinPoint detector were less than 3% (less than 0.5 mm) for the SFD, microDiamond, and Semiflex 3D detectors. For the rest of the field sizes, all the differences between the parameters measured with the different detectors were less than 3%, except for the R_100_ which differed by up to 10% (1.5 mm) for the detectors with the lowest active volume (SFD and microDiamond).

## Discussion

Firstly, with respect to OFs determination, by the time we were commissioning our 6 MV/6 MV FFF Versa HD, the piece of literature related with this topic was still somewhat heterogeneous. To our knowledge, only Lechner *et al*. work [27] was sufficiently systematic by covering a wide set of detectors and by reporting a complete series of corrections. This publication was really useful for us to notice detector behaviour and led some of our decisions when providing input for our therapy planning system. However, we decided to use our raw estimates for OFs until an institutional response like an IAEA's code of practice was available. As mentioned before, it has come while preparing this manuscript, so we decided to keep our first determinations, which are representative of what users traditionally did in the absence of calculations of correction factors, and also present the real OFs in the way TRS 483 establishes [18] along with their uncertainties as a basis for a novel comparison.

The major differences between the detector responses is caused by their volumes. Our homogeneous set of measurements showed that MOSFET, microDiamond, and diodes are good detectors for small field dosimetry and that these can be complemented with radiochromic film verification, as shown by previously published data [39, 40]. For all the detectors analysed, we found the same general trend regardless the type of filtration used.

With the section of our study related to TRS 483 correction factors we have found that applying the $k_{Q_{clin}{,Q}_{msr}}^{f_{clin},f_{msr}}$ to our measurements makes each set of OFs (microDiamond’s and SFD’s) compatible with the other. EFD results seem to have a worse behaviour due to the lack of reproducibility of our detector.

Another result derived from our comparison of both sets of data for OFs determination is related with the so-called daisy chaining procedure to ideally minimize OFs error along a broad range of field sizes. This method, initially investigated for mitigating different over-response of silicon detectors to field size changes by Dieterich and Sherouse [31], has been also addressed by the IAEA's code of practice as mentioned above. However, this procedure does not always bring our ratios of detector readings closer to the true OFs calculated with the TRS 483 corrections. As a consequence, we would discourage other users from relying on daisy chaining and recommend instead use of Eq 1 (see materials and methods section) with IAEA's code of practice factors.

Secondly, with regard to profiles acquisitions, the penumbra in the crossline direction (direction of the leaves) was greater than in the jaw direction (inline) for every field size. This difference is caused by higher transmission through the rounded MLC leaves [36]. As seen in our results, high-resolution diodes and small-volume ICs and detectors help to accurately measure the penumbra in these small fields [37,41].

Finally, with respect to percentage depth doses determination, the R_100_ of equivalent-quality FFF beams was higher compared to the corresponding flattened beams. This effect was explained by Huang et al., [42] who reported that the R_100_ shift was influenced by two competing processes: the increased contribution of low-energy photons caused by removing the flattening filter (upstream R_100_ shift), and the increased number of penetrating photons resulting from the increased beam quality (downstream R_100_ shift). The combined effect of these two competing processes results in a deeper R_100_ for equivalent-quality FFF beams.

## Conclusions

There were no substantial differences in the dose responses for FF and FFF beams that could have any clinically relevant consequences for any of the detectors investigated. Both the results of the OF and for the profiles and PDDs are clearly consistent with previously published data relating to the Versa HD, and thus these findings will help other professionals who are commissioning new Versa HD linacs. These data provide valuable insight into accurate beam modelling, which in turn, determines treatment outcomes and patient safety.

Using newly available TRS 483 corrections provide more consistent sets of results for OF determination that daisy chaining procedures. Correcting our first OFs, taken as ratio of detector charges, with the IAEA's TRS 483 corrections to obtain the final OFs, did not make the former significantly different.

## Supporting information

S1 File OFreference.xls. (XLS)

S2 File OFreference0.6-1.xls. (XLS)

S3 File Uncertainty.xlsx. (XLSX)
